# Identification and Characterization of USP7 Targets in Cancer Cells

**DOI:** 10.1038/s41598-018-34197-x

**Published:** 2018-10-26

**Authors:** Anna Georges, Edyta Marcon, Jack Greenblatt, Lori Frappier

**Affiliations:** 1 Department of Molecular Genetics, University of Toronto, Toronto, Ontario Canada; 2 Donnelly Centre, University of Toronto, Toronto, Canada

**Keywords:** Ubiquitylated proteins, Tumour-suppressor proteins, Protein-protein interaction networks

## Abstract

The ubiquitin specific protease, USP7, regulates multiple cellular pathways relevant for cancer through its ability to bind and sometimes stabilize specific target proteins through deubiquitylation. To gain a more complete profile of USP7 interactions in cancer cells, we performed affinity purification coupled to mass spectrometry to identify USP7 binding targets in gastric carcinoma cells. This confirmed reported associations of USP7 with USP11, PPM1G phosphatase and TRIP12 E3 ubiquitin ligase as well as identifying novel interactions with two DEAD/DEAH-box RNA helicases, DDX24 and DHX40. Using USP7 binding pocket mutants, we show that USP11, PPM1G, TRIP12 and DDX24 bind USP7 through its TRAF domain binding pocket, while DHX40 interacts with USP7 through a distinct binding pocket in the Ubl2 domain. P/A/ExxS motifs in USP11 and DDX24 that are critical for USP7 binding were also identified. Modulation of USP7 expression levels and inhibition of USP7 catalytic activity in multiple cells lines showed that USP7 consistently stabilizes DDX24, DHX40 and TRIP12 dependent on its catalytic activity, while USP11 and PPM1G levels were not consistently affected. Our study better defines the mechanisms of USP7 interaction with known targets and identifies DDX24 and DHX40 as new targets that are specifically bound and regulated by USP7.

## Introduction

The turnover and function of many proteins is regulated by ubiquitylation, often leading to proteasomal degradation. Ubiquitin Specific Proteases (USPs) reverse this ubiquitylation resulting in protein stabilization. USP7 (also called HAUSP) has been shown to regulate the stability of a variety of cellular proteins playing important roles in DNA damage responses, apoptosis, immune responses, DNA replication and transcription^1–4^. It is also a key regulator of the p53 pathway; binding and stabilizing the p53 E3 ubiquitin ligases Hdm2 and HdmX to downregulate p53 under normal growth conditions, but also binding and stabilizing p53 in response to DNA damage^5,6^. In addition to its role in cleaving ubiquitin chains targeting proteins for degradation, USP7 can affect protein localization and function by reversing monoubiquitylation^7–10^. Due to its multiple roles in processes that impact viral infection, USP7 is targeted by proteins from several DNA viruses, particularly herpesviruses, enabling these viruses to evade antiviral responses and replicate efficiently^9,11–22^.

USP7 has been shown to use two different binding pockets to recognize its target proteins, both of which are distinct from its central catalytic domain. The first binding pocket is within the N-terminal TRAF domain. This pocket was first identified as binding p53, Hdm2, Hdmx and the Epstein-Barr virus (EBV) EBNA1 protein^16,23–25^, and later shown to also mediate binding to minichromosome maintenance binding protein (MCM-BP)^26^, F-box protein FBXO38^27^, telomeric shelterin component TPP1^28^, ubiquitin E2 UbE2E1^29^ and the vIRF1 and vIRF4 proteins of Kaposi’s sarcoma associated herpesvirus (KSHV)^14,22^. Structures and mutational analysis showed that a P/A/ExxS motif in all of these proteins mediated the interaction with the TRAF domain binding pocket and that USP7 amino acids D164 and W165 in this pocket are essential for mediating these interactions^16,22–26^. The second binding pocket in USP7 is within one of the ubiquitin-like structures (Ubl2) in the C-terminal domain. This pocket is bound by GMP synthetase (GMPS), DNMT1, UHRF1, RNF169 and the herpes simplex virus 1 (HSV-1) protein ICP0, and involves an interaction of KxxxK motifs in these proteins with USP7 amino acids D762 and D764^30–35^.

Proteomic based studies on USP7^36^, as well as individual specific studies, have identified numerous proteins that bind USP7. However, in many cases the mechanism by which USP7 binds these proteins and whether or not this binding stabilizes these proteins has not been determined. For example, USP7 has been reported to associate with USP11, leading to regulation of polycomb repressive complex 1 (PRC1), but little is known about the mechanism of this interaction^36,37^. Like USP7, USP11 appears to have multiple cancer-associated roles, that could either promote or suppress oncogenesis^38–42^ and therefore a better understanding of the USP7-USP11 interaction is warranted. An interaction of USP7 with the ATM-dependent phosphatase PPM1G has also been reported but not well characterized^36,43^. PPM1G has been reported to dephosphorylate USP7 in response to DNA damage, leading to downregulation of USP7 levels^43^. However, the mechanism of this interaction and whether or not USP7 reciprocally regulates PPM1G protein levels has not been determined.

In keeping with the roles of USP7 in regulating several cancer-related pathways, upregulation of USP7 in several cancers has been shown to promote oncogenesis, identifying USP7 as an attractive target for cancer therapy^7,44–48^. Accordingly, several USP7-specific inhibitors have been recently developed^49–55^. However, to better understand the contributions of USP7 to cancer, a more thorough understanding of its interactions in cancer cells is needed. Here we use affinity purification coupled to mass spectrometry (AP-MS) to identify USP7 interactions in gastric carcinoma cells. We then determine the USP7 binding pocket used to interact with several of the uncharacterized or poorly characterized USP7 targets (specifically DEAD-box protein DDX24, DEAH-box helicase DHX40, USP11 and PPM1G), as well as the effect of USP7 on these protein levels. In addition, we identify the USP7 binding sites on USP11 and DDX24, defining the mechanism of the USP7 interaction.

## Results

### Identification of USP7 interactors in gastric carcinoma and nasopharyngeal carcinoma cells

To gain a better understanding of USP7 interactions in cancer, we expressed FLAG-tagged USP7 in AGS gastric carcinoma cells and performed AP-MS experiments, in which USP7 and associated proteins were recovered on anti-FLAG resin and recovered proteins were identified by tandem mass spectrometry (LC-MS/MS). USP7-FLAG was delivered to the cells by adenovirus infection at titres resulting in expression levels close to endogenous USP7. To eliminate nonspecific interactions, total spectral counts of each protein recovered with USP7 were compared to that with the β-GAL-FLAG negative control, as well as to the Containment Repository for Affinity Purification (http://CRAPome.org), a database of interactors from over 400 AP-MS experiments. The top USP7 interactions identified in two independent experiments are shown in Table 1 and the complete interaction data is provided in Supplemental Table S1. Consistent with previous reports, the USP7 interactors included USP11^36,37,56^, PPIL4^36^, GMP synthetase (GMPS)^9,10,32^, PPM1G phosphatase^36,43^, FBXO38^27^, TRIP12 E3 ubiquitin ligase^57,58^ and DNMT1^59–61^. In addition, we identified previously unreported interactions with two DEAD/DEAH-box RNA helicases, DDX24 and DHX40, as well as with Melanoma-associated antigen D2 (MAGED2), coiled coil domain-containing protein 55 (CCDC55) and Transcription Elongation Factor A Like 4 (TCEAL4). DDX24 has several important roles in RNA-mediated innate immune signaling, p53 regulation and human immunodeficiency virus type-1 (HIV-1) infection^62–64^, while DHX40 is largely uncharacterized. We chose to further investigate the novel interactions of USP7 with DDX24 and DHX40, along with the poorly characterized interactions with USP11 and PPM1G. TRIP12 was also included in our study as an example of a characterized USP7 interactor that binds through the USP7 TRAF domain^57,58^.

**Table 1:** Affinity Purification-Mass Spectrometry Results for FLAG-USP7.

Identified proteins	Total Spectral Counts*			
	Protein ID	FLAG-USP7	FLAG-β GAL	CRAPome (Average)
USP7	Q93009	1010|922	3|2	
USP11	P51784	98|122	0|0	3.2
DDX24	Q9GZR7	73|57	0|0	3.4
PPIL4	Q8WUA2	62|60	0|0	4.7
MAGED2	Q9UNF1	52|24	0|0	2.7
GMPS	P49915	50|48	0|0	3.6
PPM1G	O15355	49|22	0|0	2
DHX40	Q8IX18	27|33	0|0	2
CCDC55	Q9H0G5	19|17	0|0	2.1
TCEAL4	Q96EI5	19|18	0|0	2
FBXO38	Q6PIJ6	18|32	0|0	0
TRIP12	Q14669	17|16	0|0	4.3
DNMT1	P26358	5|20	0|0	3.7

*Total spectral counts from two independent experiments are shown (separated by a line).

**Figure 1:**
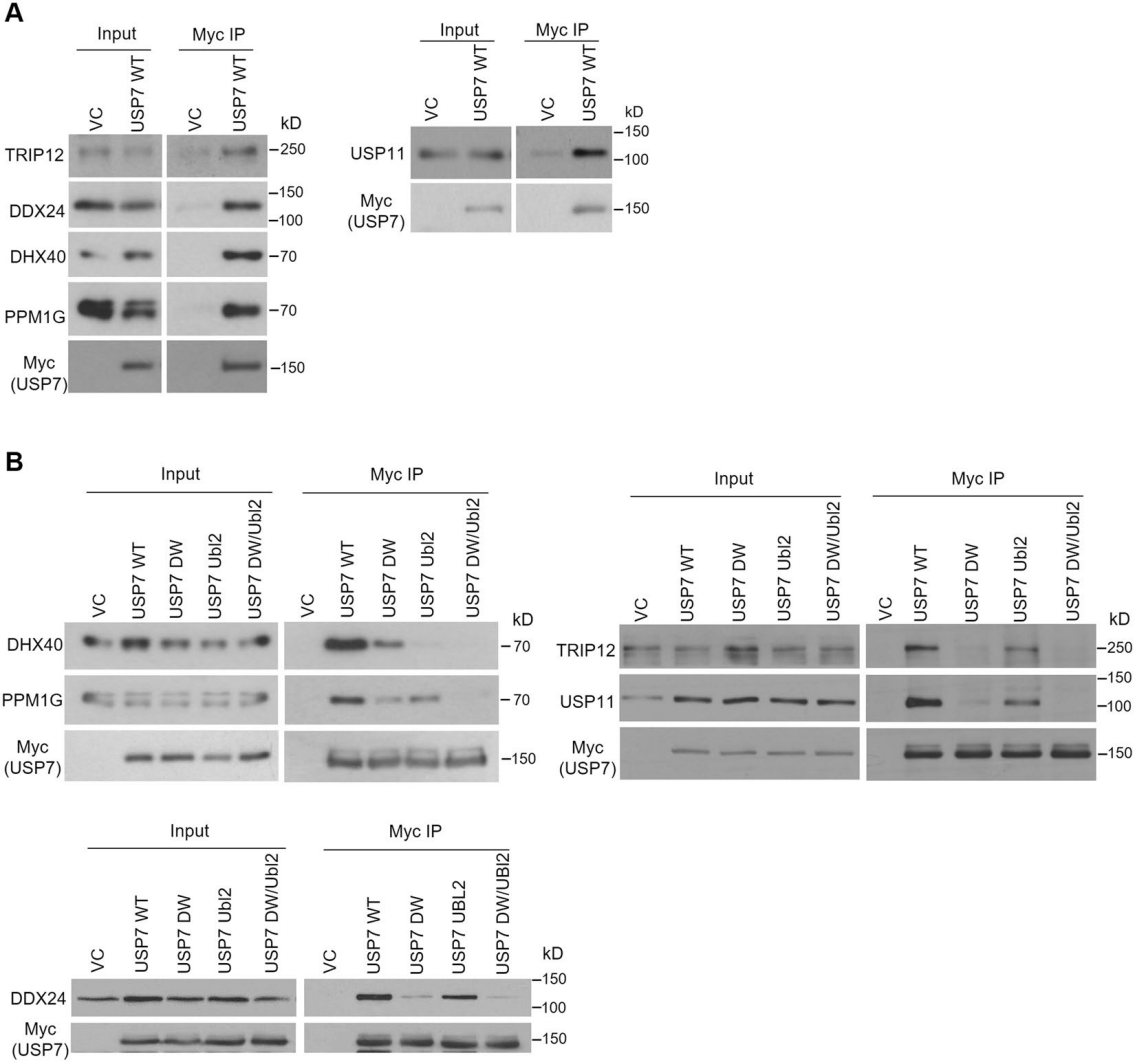
Coimmunoprecipitation of USP7 target proteins with WT and binding pocket mutations of USP7. (**A**) AGS cell were transfected with a plasmid expressing myc-tagged USP7 or an empty myc plasmid control (VC). Myc-USP7 was immunoprecipitated with anti-myc resin and recovered proteins were analyzed by Western blotting using antibodies against myc and the indicated endogenous proteins. (**B**) CNE2Z cells were transfected with empty vector control (VC) plasmid or plasmids expressing myc-tagged USP7 with WT sequence or with mutations in the TRAF (DW), or Ubl2 binding pocket or both binding pockets (DW/Ubl2). Myc-USP7 was recovered by Myc immunoprecipitation, followed by Western blotting as in (**A**).

We first validated the interactions of USP7 with USP11, PPM1G, DHX40, DDX24 and TRIP12 in AGS cells by transiently expressing myc-tagged USP7 (or empty myc plasmid) followed by myc immunoprecipitation and Western blotting for the endogenous proteins as shown in Fig. 1A (full length Western blots for all figures are provided in Supplementary Fig. S1). This confirmed that USP11, PPM1G, DHX40, DDX24 and TRIP12 were all specifically recovered with USP7. We then repeated this experiment in a nasopharyngeal carcinoma cell line (CNE2Z) to determine if these interactions occurred in the context of other cancer cell lines. As shown in Fig. 1B, USP11, PPM1G, DHX40, DDX24 and TRIP12 were all recovered with myc-USP7 but not with myc alone, confirming these USP7 interactions in this cell background.

### Identifying binding sites on USP7

USP7 is known to contain two binding pockets that recognize specific target proteins; one in the N-terminal TRAF domain involving contacts with residues D164 and W165, and a second in the C-terminal Ubl2 domain involving contacts with D762 and D764^16,22–26,30^. To determine which binding pocket was used to mediate the interactions with USP11, PPM1G, DHX40, DDX24 and TRIP12, we repeated the myc-USP7 co-IP experiments in CNE2Z cells, using USP7 mutants with D164A,W165A mutations to disrupt the TRAF binding pocket (referred to as DW) or D762R,D764R mutations to disrupt the Ubl2 pocket (referred to as Ubl2). We also combined these mutations to disrupt both binding pockets (referred to as DW/Ubl2). As shown in Fig. 1B, the DW/Ubl2 mutations disrupted binding to all of the tested USP7 target proteins, indicating that all of the proteins bind to one of the two previously defined USP7 binding pockets. Comparison of protein recoveries with individual DW and Ubl2 USP7 mutants showed that interactions with PPM1G, USP11, DDX24 and TRIP12 were greatly affected by the DW mutation and much less affected by the Ubl2 mutation, indicating that these proteins all interact predominantly bind USP7 through the TRAF binding pocket. The results with TRIP12 are consistent with previous reports that TRIP12 binds the TRAF domain of USP7^58^. In contrast, the DHX40 interaction was abrogated by the Ubl2 mutation and only partly affected by the DW mutation, indicating that this protein binds predominantly through the Ubl2 pocket.

### Mapping the USP7 binding site on USP11

**Figure 2:**
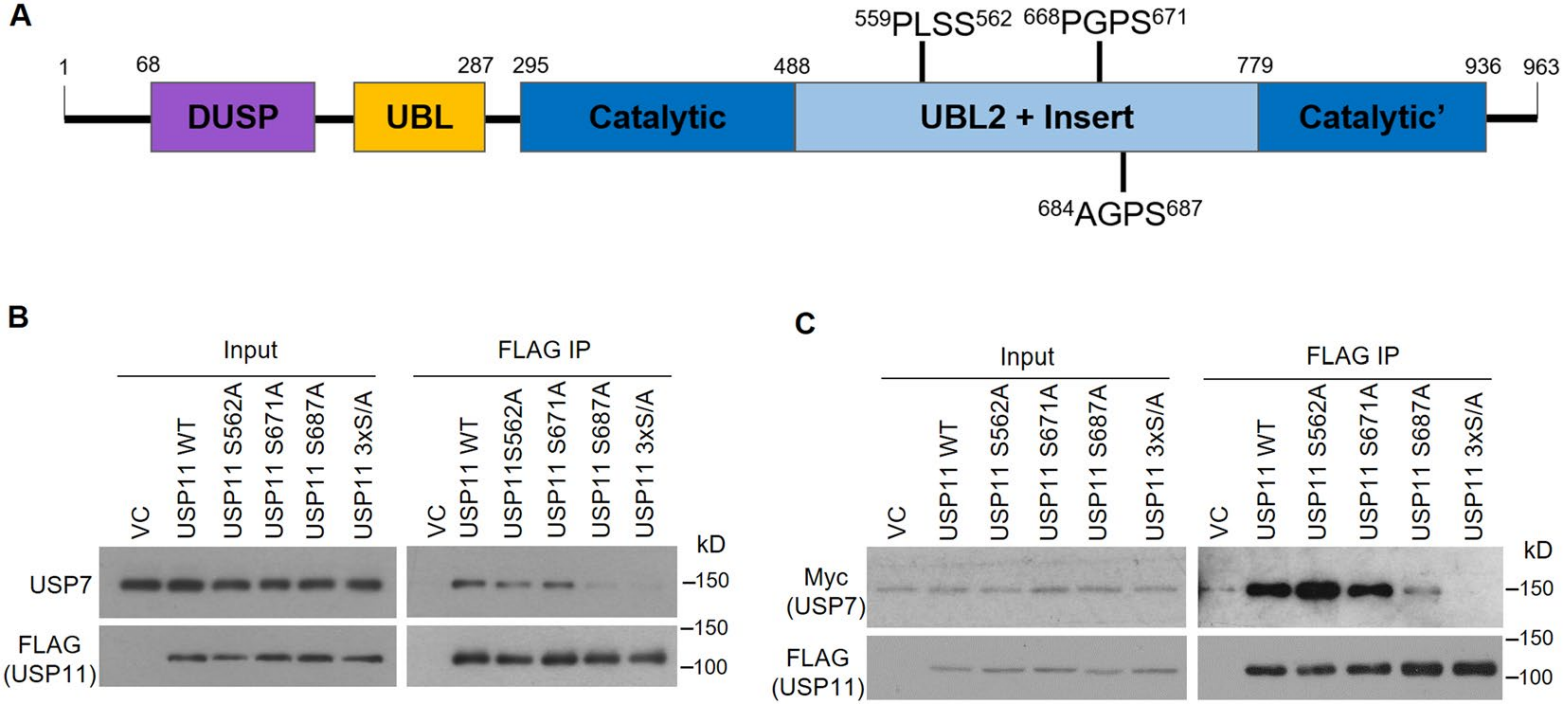
Identification of the USP7 binding site in USP11. (**A**) Schematic representation of the USP11 protein indicating the location of the P/AxxS motifs that were mutated in this study. DUSP: domain present in ubiquitin specific proteases; UBL: ubiquitin-like domain. (**B**) AGS cells were transfected with empty FLAG plasmid (VC) or plasmids expressing FLAG-tagged USP11 with WT sequence or the indicated point mutations or triple mutation (S562A/S671A/S687A; 3xS/A). FLAG-tagged proteins were immunoprecipitated with anti-FLAG resin and recovered proteins were analyzed by Western blotting using antibodies against FLAG and USP7. (**C**) CNE2Z cells were co-transfected with plasmids expressing Myc-tagged USP7 and the set of FLAG-tagged USP11 constructs as in (A) or with empty FLAG plasmid (VC). FLAG-tagged proteins were immunoprecipitated as in (**A**) and recovered proteins were analyzed by Western blotting using antibodies against FLAG and myc.

Since USP7 and USP11 are reported to have a functional connection^36,37^, we further investigated the mechanism of this interaction. The above experiments showed that USP11 binds USP7 through the TRAF binding pocket. We previously identified a P/A/ExxS motif in other USP7 binding partners that mediates the interaction with the TRAF binding pocket, and showed that mutating the serine in this motif to alanine is sufficient to disrupt the interaction^16,23,25^. Therefore we searched USP11 for this motif. USP11 contains an insert in the middle of its catalytic domain consisting of a Ubl and additional sequences^65^. Three P/A/ExxS motifs (^559^PLSS^562^, ^668^PGPS^671^ and ^684^AGPS^687^) were present in this insert (as shown in Fig. 2A), representing potential USP7 binding sites. We changed the S to A in each of these motifs individually to generate S562A, S671A and S687A point mutants and also combined these three mutations to generate a S562A/S671A/S687A (3xS/A) triple mutant. We then expressed FLAG-tagged USP11 containing either WT sequence or the mutations in AGS cells, followed by immunoprecipitation with anti-FLAG resin and Western blotting for USP7 (Fig. 2B). We also co-expressed myc-USP7 with FLAG-tagged USP11 containing either WT sequence or the mutations followed by FLAG-IP and Western blotting for myc. In both cases, USP7 binding by USP11 was greatly decreased by the S687A mutation but not the S562A and S671A mutations. In addition, USP7 binding was abrogated by the triple (3xS/A) mutation. These results indicate that the USP11 ^684^AGPS^687^ motif is the major site recognized by USP7 (although in its absence there may be some propensity to interact with ^559^PLSS^562^ and ^668^PGPS^671^).

### Mapping the USP7 binding site on DDX24

**Figure 3:**
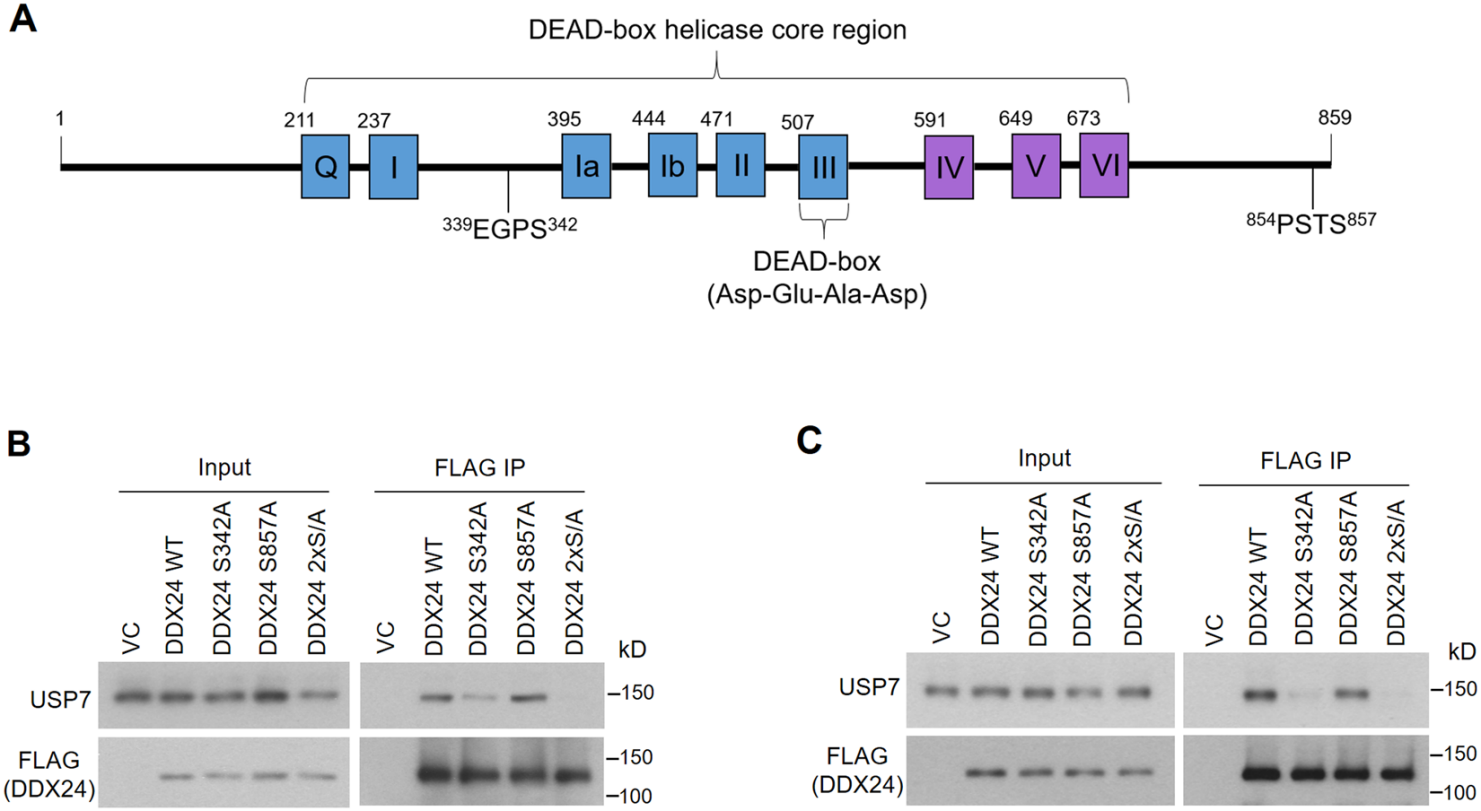
Identification of the USP7 binding site in DDX24. (**A**) Full length DDX24 protein sequence indicating the two P/ExxS (^339^EGPS^342^ and ^854^PSTS^857^) motifs that were mutated in this study (underlined) (**B**,**C**) AGS cells (**B**) and CNE2Z cells (**C**) were transfected with an empty FLAG plasmid control (VC) or plasmids expressing FLAG-tagged DDX24 with WT sequence or S342A, S857A or S342A/S857A (2xS/A) mutations. FLAG-tagged proteins were immunoprecipitated using anti-FLAG resin and recovered proteins were analyzed by Western blotting using antibodies against FLAG and USP7.

DDX24 is known to have several important functions but its interaction with USP7 has not been previously investigated. Since we found that DDX24 bound to the USP7 TRAF binding pocket, we examined DDX24 for P/A/ExxS motifs that typically mediate this interaction. Two of the 15 putative P/A/ExxS motifs in DDX24 (^339^EGPS^342^ and ^854^PSTS^857^; Fig. 3A) are identical to previously characterized USP7 binding sites. Namely, ^339^EGPS^342^ is identical to the EGPS motifs that mediate USP7 binding in EBV EBNA1 and KSHV vIRF1^22,29^, and ^854^PSTS^857^ is identical to the PSTS USP7 binding sequences in Hdm2 and MCM-BP^23,25,26^. Therefore we tested whether these two motifs mediated DDX24 binding to USP7 by generating S342A and S857A point mutations as well as a S342A/S857A double mutant (2xS/A). FLAG-DDX24 containing these mutations or WT sequence was expressed in AGS and CNE2Z cells, followed by FLAG immunoprecipitation and Western blotting for endogenous USP7 (Fig. 3B,C). DDX24 binding to USP7 was abrogated in the S342A/S857A double mutant, greatly decreased in the S342A single mutant and unaffected in the S857A mutant. These results indicate that the ^339^EGPS^342^ sequence of DDX24 is the main site of interaction with USP7.

### USP7 protects DDX24 and DHX40 from proteasomal-mediated degradation

**Figure 4:**
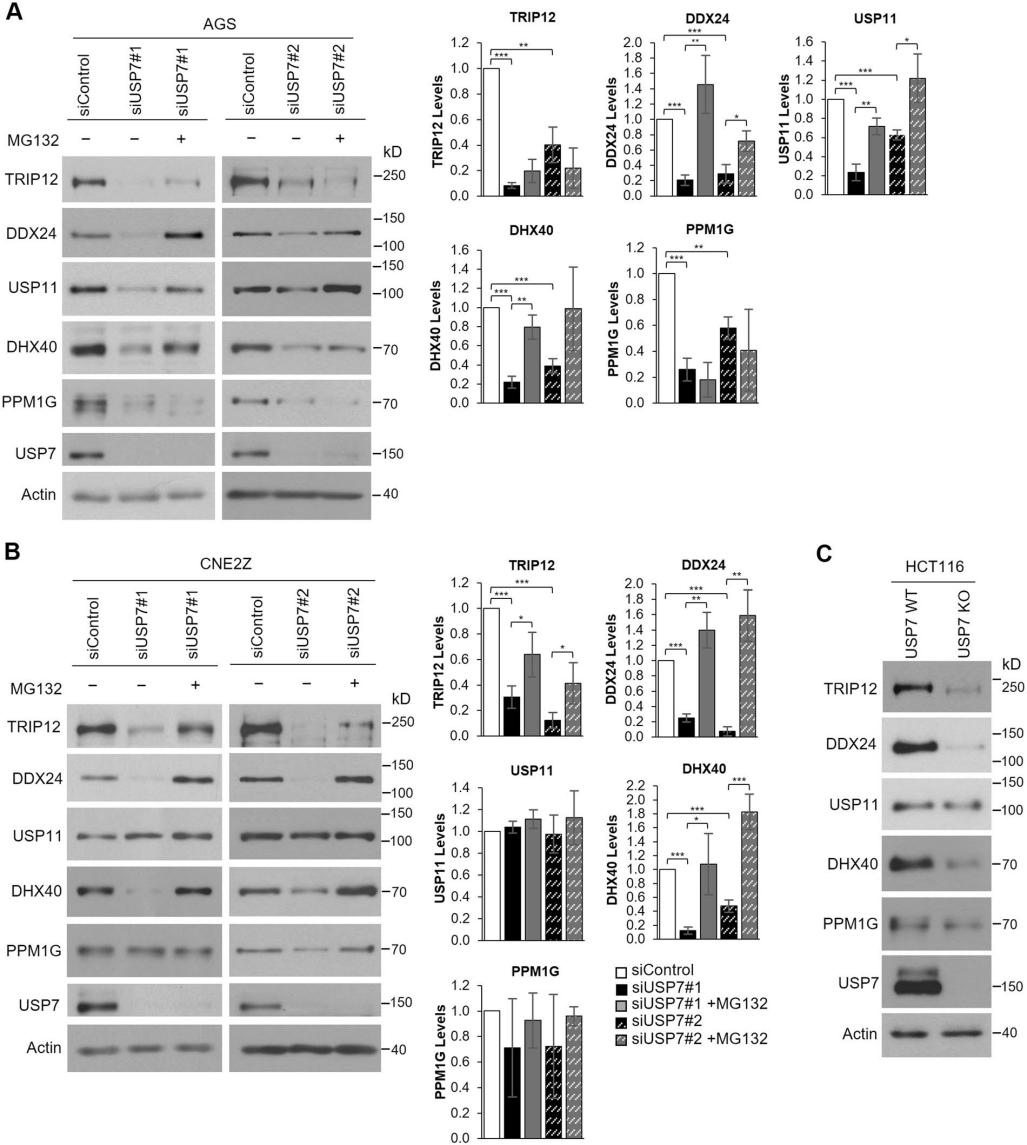
Effects of USP7 depletion on target protein levels. (**A**,**B**) AGS (**A**) or CNE2Z (**B**) cells were transfected with two different siRNAs targeting USP7 (#1 or #2) or a negative control siRNA (siControl) followed by treatment with the MG132 proteasome inhibitor (+) or DMSO as a negative control (−). Cell lysates were analyzed by Western blotting using the indicated antibodies. For each condition, the protein bands for TRIP12, DDX24, USP11, DHX40 and PPM1G were quantified in three independent USP7 silencing experiments (+/−MG132) and normalized to actin. The bar graphs to the right of the Western blots show the average values relative to the silencing control for each protein. P values for siUSP7#1 or 2 are indicated relative to siControl and p values for siUSP7#1/2 + MG132 are indicated relative to siUSP7#1/2 without MG132 (*0.01 < *P* < 0.05; **0.001 < *P* < 0.01; ***P < 0.001). (**C**) Whole cell lysates of HCT116 cells (WT) or HCT116 with USP7 knockout (KO) were analyzed by Western blotting as in (**A**).

USP7 is known to deubiquitylate and stabilize many of its binding partners. To investigate whether the USP7 interactors that we identified were stabilized by USP7, we first silenced USP7 with two different siRNAs in both AGS and CNE2Z cells and performed Western blots on endogenous DDX24, DHX40, PPM1G, USP11 and TRIP12 to determine whether their levels were decreased, as expected if they were stabilized by USP7. In addition, to determine whether any decrease in protein levels was due to proteasomal-mediated degradation (as expected if stabilization by USP7 is due to deubiquitylation) one set of USP7-silenced samples was treated with the proteasome inhibitor, MG132. Sample experiments and quantification of the bands from multiple experiments are shown in Fig. 4A (AGS cells) and 4B (CNE2Z cells). The results show that the levels of DDX24 and DHX40 were greatly decreased by USP7 silencing in both cell lines and that these levels were restored with MG132 treatment. Similarly TRIP12 was consistently decreased by USP7 silencing, although in most cases there was little to no rescue of the levels with MG132 treatment. This may indicate that the autophagy-lysosomal pathway plays a more prominent role in TRIP12 degradation than the proteasomal pathway. The levels of USP11 and PPM1G were inconsistently affected by USP7 silencing; both proteins were decreased upon USP7 silencing in AGS cells but not in CNE2Z cells. Since USP11 levels in AGS cells were restored by MG132 treatment, the difference in USP7 effects in the two cell lines might reflect different turnover rates of USP11 in the two cell backgrounds, such as could be caused by different levels of USP11-targeted ubiquitin ligases. However, the decreased PPM1G levels in AGS cells after USP7 silencing were not affected by MG132, suggesting that any effects of USP7 silencing were not due to proteasomal-mediated degradation, but rather could involve autophagy-lysosomal pathway degradation or an indirect effect on PPM1G expression.

In addition, we compared the levels of DDX24, DHX40, PPM1G, USP11 and TRIP12 in HCT116 cell lines with and without a USP7 knockout. Consistent with the silencing experiments, DDX24, DHX40 and TRIP12 levels were all greatly decreased in the USP7 knockout cells, whereas the levels of USP11 and PPM1G were not obviously affected. Together these results indicate that USP7 binds and stabilizes DDX24, DHX40 and TRIP12 but that stabilization of USP11 is only evident in specific cell backgrounds.

Since, USP11 and TRIP12 can also affect protein stability through ubiquitylation, we examined whether these proteins might affect the stability of USP7. To this end, we silenced USP11 or TRIP12 in AGS and CNE2Z cells and performed Western blots for endogenous USP7 (Supplementary Fig. S1). We found no obvious effect on USP7 levels, suggesting that these proteins are not major factors in regulating the turnover of USP7. The TRIP12 result is consistent with previous reports that showed that knockdown of TRIP12 by shRNA does not affect USP7 levels in hepatocellular carcinoma cells^57^.

### The deubiquitylating activity of USP7 is required to stabilize DDX24, DHX40 and TRIP12

**Figure 5:**
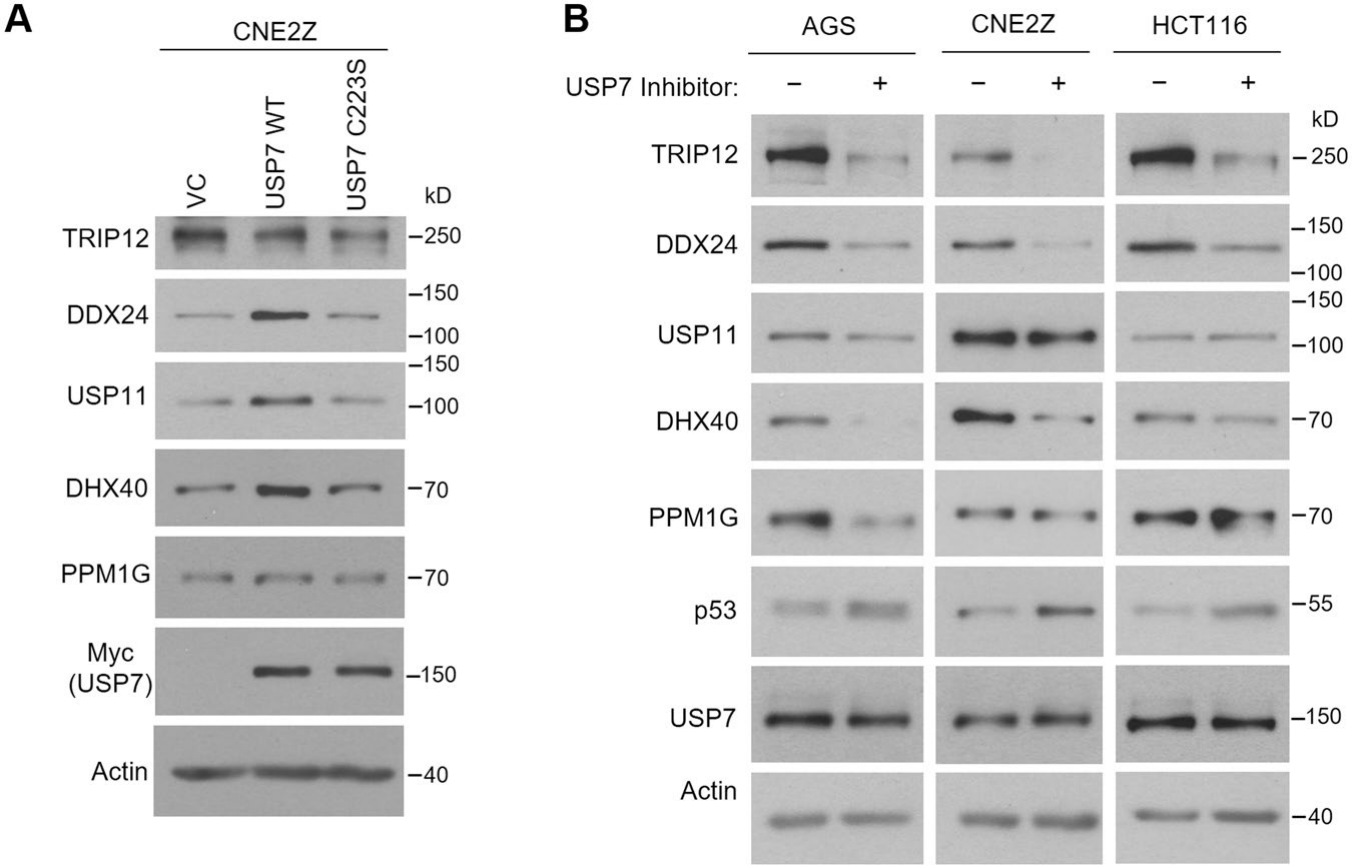
The role of USP7 catalytic activity in stabilizing target proteins. (**A**) CNE2Z cells were transfected with plasmids expressing myc-USP7 with WT sequence or C223S mutation or with an empty vector control (VC). 48 hrs later, cell lysates were analyzed by Western blotting using the indicated antibodies. (**B**) AGS, HCT116 or CNE2Z cells were treated with 5 µM of compound 4 (USP7 inhibitor) or an inactive enantiomer and harvested after 8 hours (CNE2Z) or 24 hours (AGS and HCT116). The shorter time was necessary in CNE2Z cells due to toxicity. Whole cell lysates were analyzed by Western blotting using the indicated antibodies.

We next tested whether the stabilization of target proteins by USP7 that we observed required the deubiquitylation activity of USP7. We addressed this question in two ways. First, we determined whether overexpression of USP7 stabilized the proteins dependent on its catalytic cysteine (C223). Overexpression of WT USP7, but not the C223S catalytically inactive mutant of USP7, was found to increase the levels of DDX24, DHX40 and USP11 (Fig. 5A). Consistent with the USP7 silencing experiments, PPM1G levels were not affected by USP7 overexpression. Surprisingly, TRIP12 levels, which were affected by USP7 silencing, were unchanged upon overexpression of USP7 WT. This might indicate that endogenous USP7 levels are already in excess over TRIP12 levels.

In the second approach to assess the importance of the USP7 deubiquitylation activity for protein stabilization, we treated cells with a highly specific inhibitor of USP7 catalytic activity (Compound 4, also known as AD04) recently developed by Harrison *et al*.^50^ or an inactive enantiomer of this compound, and examined effects on the level of endogenous USP7 target proteins. The experiment was performed in three cell lines (AGS, CNE2Z and HCT116) and results are shown in Fig. 5B. p53 was examined as a positive control for USP7 inhibition, which is known to result in increased p53 levels due to destabilization of the p53 E3 ubiquitin ligases, Hdm2 and Hdmx^5,50,66,67^. p53 levels were increased by compound 4 in all three cell lines, confirming its inhibition of USP7 (Fig. 5B). Consistent with the USP7 silencing and knockout experiments, the USP7 inhibitor resulted in decreased levels of DDX24, DHX40 and TRIP12 in all three cell lines, whereas PPM1G and USP11 levels were only decreased by the inhibitor in AGS cells. Together, the results indicate that USP7 stabilizes DDX24, DHX40 and TRIP12 by deubiquitylation in multiple cell lines, whereas stabilization of USP11 and PPM1G by USP7 varies in different cell backgrounds.

## Discussion

Through studies of its numerous binding partners and substrates, USP7 has emerged as a key regulator of many important cellular process and oncogenic pathways. Here we use a proteomics approach to further identify specific USP7 targets in the context of gastric carcinoma cells. This confirmed some previously reported functional USP7 interactions, including one with TRIP12 E3 ubiquitin ligase, that we further showed binds USP7 through the TRAF binding pocket and is stabilized by USP7. In addition, we identified novel interactions with two DEAD/DEAH box RNA helicases, DDX24 and DHX40, both of which are stabilized by USP7 dependent on its deubiquitylating activity. We further show that USP7 binds these proteins through two different binding pockets; the DDX24 interaction is mediated by the TRAF domain binding pocket, while the DHX40 interaction occurs predominantly through the Ubl2 binding pocket.

Experiments involving USP7 depletion, overexpression and inhibition of its catalytic activity in multiple cell lines, all indicated that USP7 stabilizes DDX24 and DHX40 through its catalytic activity, preventing their proteasomal degradation. This suggests that USP7 can regulate the functions associated with these proteins by affecting their abundance. DExH/D-box RNA helicases are ATP-dependent RNA helicases that play important roles in many RNA processes, including ribosome biogenesis, pre-mRNA splicing, RNA export and RNA turnover^68,69^. The functional roles of DHX40 in RNA processes have yet to be identified, although it has been reported to be similar to human DDX8, as well as Prp22 and Dhr1 from *Saccharomyces cerevisiae*^70^. DHX40 is also ubiquitously expressed in a wide variety of tissues, suggesting it has a general function in RNA metabolism^70^. While most of the protein targets that are stabilized by USP7 are bound through the USP7 TRAF domain, we find that DHX40 is preferentially bound through the USP7 Ubl2 binding pocket. DHX40 thus joins a short list of USP7 interactors that bind through this site, including GMPS, UHRF1, DNMT1, RNF169 and the HSV-1 protein ICP0^30,34,35^. We have previously shown that this USP7 pocket recognizes KxxxK motifs^30^, and the DHX40 protein sequence contains four such motifs that might be responsible for this interaction.

In contrast to DHX40, several functions have been identified for DDX24. These include a role in regulating pre-rRNA processing^71^ as well as a role in the interferon pathway as a negative regulator of RIG-I-like receptors^64^. The ability of USP7 to stabilize DHX40 suggests that USP7 may also impact these processes. In addition, DDX24 was recently reported to inhibit the transcriptional activity of the p53 tumor suppressor by antagonizing its acetylation^62^. As a result, DDX24 negatively regulates p53-dependant cell cycle arrest and senescence and promotes tumor cell growth. This is consistent with reports that DDX24 depletion inhibits the growth of multiple cancer cell lines and that its overexpression correlates with a lower survival rate in gastric and HER2-positive breast cancer patients^72^. USP7 is also known to regulate p53 activity; by deubiquitylating and stabilizing p53 in response to DNA damage or by destabilizing p53 through stabilization of Hdm2 and Hdmx under normal growth conditions^5,6^. Our findings that USP7 stabilizes DDX24 suggests an additional mechanism by which USP7 interferes with p53 activity and contributes to oncogenesis. Finally, DDX24 has been found to contribute to HIV-1 infectivity by interacting with the HIV-1 Rev protein and affecting mRNA nuclear export^63^. USP7 has also been shown to promote HIV-1 infection by deubiquitylating and stabilizing the HIV-1 Tat protein^73^. Our studies suggest that USP7 may play an additional role in HIV-1 infection through its stabilization of DDX24.

We have investigated the mechanism of the USP7-DDX24 interaction and shown that USP7 binds DDX24 predominantly through its TRAF binding pocket. We have previously shown that the USP7 TRAF domain recognizes P/A/ExxS motifs in target proteins, with the EGPS motif that occurs in EBV EBNA1 and KSHV vIRF4 proteins being the strongest interactions so far identified^16,22^. Here we show that amino acids 339–342 of DDX24 also contains an EGPS motif and that mutation of S342 disrupts USP7 binding. DDX24 is the first cellular protein found to bind USP7 using an EGPS motif, and the interaction through this motif suggests a high affinity direct interaction that would effectively compete with many other cellular protein interactions for this USP7 site.

Our proteomics experiments also identified physical interactions of USP7 with USP11 and PPM1G; associations which were known but not well characterized. Like the DDX24 interaction, we showed that interactions with USP11 and PPM1G occurred predominantly through the USP7 TRAF domain. PPM1G was previously reported to bind and dephosphorylate USP7 in response to DNA damage, leading to decreased USP7 levels^43^. However, the fact that we identified the USP7-PPM1G interaction under normal cell growth conditions, suggests that this interaction may have additional functions that are independent of the DNA damage pathway. This could involve PPM1G-mediated dephosphorylation of USP7 but might also involve USP7-mediated relocalization of PPM1G to other substrates to impact its many functions, including regulation of cell cycle progression^74^, transcription^75,76^ and translation^77,78^.

For USP11, we identified ^684^AGPS^687^ as the major site that mediates the interaction with USP7. The USP7 substrate TPP1 has also been reported to bind USP7 using an AGPS motif^28^. Since the AGPS sequence matches P/A/ExxS motifs known to bind USP7 directly, our results suggest that USP7 binds directly to USP11. USP11 ^684^AGPS^687^ is located within the C-terminal region (503–920) that mediates interactions with its targets p21, VGLL4 and XIAP^40,41,79^, raising the possibility that these interactions might physically impede each other.

Although we saw some evidence of USP7-mediated stabilization of USP11 in AGS cells, the results in CNE2Z and HCT116 cells suggest that USP7 does not consistently stabilize this protein. Similarly, we did not find evidence that USP11 stabilized USP7. This does not exclude the possibility that USP7 and USP11 could remove non-degradative ubiquitin signals from one another, that might affect protein interactions and functions. In support of this possibility, USP11 has been shown to have a preference for the non-degradative K63 ubiquitin chain linkages over degradative K48 chains^65^. Interestingly, USP11 and USP7 have been reported to have some protein targets in common, including the xeroderma pigmentosum complementation group C (XPC) protein and multiple components of the polycomb repressive-1 (PRC1) complex^37,80,81^. Furthermore, the promyelocytic leukemia protein (PML) is regulated by both USP7 and USP11, although they have opposite effects^82,83^. Our finding that USP7 and USP11 physically interact raise the possibility that they function as a complex to regulate the levels and activities of their shared binding partners. It is also possible that USP7 binding to USP11 could inhibit its catalytic activity, since the USP7 binding site falls within an insert in the USP11 catalytic domain. The USP7-binding point mutant of USP11 that we have generated (S687A) will be a useful tool to investigate the importance of USP7 binding for the various functions of USP11.

In summary, we have identified DHX40 and DDX24 as novel targets of USP7 that are both bound and stabilized by USP7, implicating USP7 as a regulator of RNA metabolism through effects on these proteins and providing an additional potential mechanism of p53 regulation by USP7. We also reveal the mechanisms of USP7 interactions with USP11, PPM1G, DHX40 and DDX24, including the development of USP7-binding point mutants of USP11 and DDX24 that will be useful for function studies.

## Materials and Methods

### Cell Lines

AGS human gastric adenocarcinoma and CNE2Z EBV-negative nasopharyngeal carcinoma cells^84^ were maintained in RPMI 1640 and alpha-MEM (Gibco), respectively and supplemented with 10% fetal bovine serum (FBS, Wisent Inc). The HCT116 human colon carcinoma cells and USP7-null HCT116 cells (obtained from Bert Vogelstein) were maintained in alpha-MEM supplemented with 10% FBS.

### Plasmids and siRNA

Plasmids expressing myc-USP7 (pCDNA3), the myc-USP7 catalytic mutant (C223S) and myc-USP7 D762R/D764R (Ubl2) were described previously^30,83^. Myc-USP7 (DW) and myc-USP7 D164A/W165A/D762R/W764A (DW/Ubl2) were gifts from Yi Sheng and Vivian Saridakis, respectively^29^. The plasmid expressing FLAG-USP11 (pCMV) was generated by PCR amplification of the USP11 sequence in a USP11 pDEST-LPCX vector (a gift from Daniel Durocher) using forward primer: 5′-GATCGGTACCACCATGGACTACAAGGACGACGATGACAAGGCAGCCATGGCAGTAGCCCCGCGA-3′and reverse primer 5′-GATCGCGGCCGCTCAATTAACATCCATGAACTCAG-3′. The resulting PCR product was inserted between the KpnI and NotI sites of the pCMV 3FC vector (previously described^85^), generating an N-terminal FLAG-tagged USP11 expression construct. The FLAG-USP11 P/AxxS mutants S562A, S671A and S687A and the triple mutant S562A/S671A/S687A (3xS/A) were generated using GeneArt^TM^ Strings^TM^ DNA Fragments (Invitrogen). The FLAG-DDX24 (pCMV) was generated by PCR amplification of the DDX24 sequence in a DDX24 pCDNA3 vector (a gift from Keiichi I . Nakayama^71^) using forward primer: 5′-CATGGGTACCACCATGGACTACAAGGACGACGATGACAAGGCAGCCATGAAGTTGAAGGACACAAAATCAAG-3′and reverse primer 5′-CATGGCGGCCGCTTAATTTGCACTTGTACTTGGCTG-3′. The resulting PCR product was inserted between the Kpn I and Not I sites of pCMV 3FC. The FLAG-DDX24 S342A (pCMV) mutant was generated using GeneArt^TM^ Strings^TM^ DNA Fragments. The FLAG-DDX24 S857A and S342A/S857A (2xS/A) mutants were generated by PCR amplification of the full length FLAG-DDX24 WT or S342A mutant, respectively, using the same forward primer described above and a reverse primer that contained the point mutant sequence: 5′-CATGGCGGCCGCTTAATTTGCTGCTGTACTTGGCTGTGGCTG -3. The resulting PCR products were inserted between the Kpn I and Not I sites of pCMV 3FC. All plasmids were verified by DNA sequencing. Stealth siRNA targeting USP7 (#1 5′-CCCAAAUUAUUCCGCGGCAAA-3′ and #2 5′-CCTCTAGCCGAAGTCTTCAGCAAGA-3′), TRIP12 (5′-GGGAUCCAUGGGAUCCACAACUUCA -3′) and USP11 were from Invitrogen. AllStar negative-control siRNA was obtained from Qiagen.

### Affinity Purification coupled to Mass Spectrometry (AP-MS)

AGS cells in15-cm diameter plates at 70% confluence were transduced with the adenoviruses (minimum amount required to infect 70% of the cells) expressing USP7 or β-Gal with C-terminal Sequential Purification Affinity (SPA) tags^86^ and harvested 48 hours post-transduction. The generation of the adenoviruses is described in Georges *et al*.^27^. Cells were lysed and extracted by the “high salt extraction” method described in Georges and Frappier^87^. The extract was incubated with 50 μl of anti-FLAG M2 resin (Sigma-Aldrich) 4 hours at 4 °C with end-over-end rotation. The resin was washed first in IPP buffer (10 mM HEPES pH 7., 100 mM NaCl, 0.1% Triton, 10% glycerol) followed by a second wash in 50 mM ammonium bicarbonate, 75 mM KCl. Bound proteins were eluted by three incubations in 150 μl 0.5 M ammonium hydroxide, pH 11. Eluates were dried by lyophilisation using a SpeedVac, washed once in high-performance liquid chromatography (HPLC)-grade water and further dried by lyophilisation. The lyophilized protein was resuspended in 50 mM ammonium bicarbonate containing 10 µg/µl proteomic grade trypsin (Sigma) and incubated overnight at 37 °C followed by further incubation for 2 hours in freshly added trypsin. Samples were lyophilised again and the peptides were analyzed by liquid chromatography-tandem mass spectrometry (LC-MS/MS) using a LTQ Orbitrap system (Thermo Finnigan) and identified using Mascot software (Matrix Science, United Kingdom).

### Transfections and USP7 inhibitor treatment

AGS or CNE2Z cells were plated in 10-cm dishes and immediately transfected with 100 pmol of small interfering RNA (siRNA) against USP7, TRIP12 or USP11 using 4 μl of Lipofectamine 2000. For USP7 and USP11 silencing, siRNA transfections were repeated two additional times after 24 and 48 h. Cells were harvested 48 h after the last round of transfection and processed for Western blotting. For proteasome inhibition, 10 µM final concentration of MG132 (Sigma) was added to the cells 12 hours before harvesting. For overexpression experiments, AGS or CNE2Z cells at ~80% confluency in 10-cm dishes were transfected with 7 µg of the indicated plasmids using transfection grade Polyethylenimine (PEI, Polyscience 23966). In brief, 7 µg of DNA was diluted in 1 ml optimum media, followed by addition of 21 µl PEI reagent. The DNA-PEI complexes were mixed briefly by vortexing and incubated at room temperature for 15 minutes prior adding to the cells. Cells were harvested 48 hours post-transfection. For the FLAG-USP11 and Myc-USP7 co-transfection experiments, 3.5 µg of each of the co-transfected plasmids were used for a total of 7 µg. For the USP7 inhibitor experiments, cells at ~40% confluency were treated with a final concentration of 5 µM of USP7 inhibitor (Compound 4; also known as AD04) or the inactive enantiomer (gifts from Timothy Harrison^50^). Cells were harvested 8 hours (CNE2Z cells) or 24 hours (AGS and HCT116 cells) post-inhibitor treatment.

### Western blotting

Cells were lysed in 9 M urea, 10 mM Tris-HCl pH 6.8, sonicated and clarified by centrifugation. 80 µg of protein was subjected to SDS-PAGE and transferred to nitrocellulose. Membranes were blocked in 5% non-fat dry milk in TBS-T (TBS with 0.1% Tween), then incubated with primary antibodies FLAG M2 (F1804 from Sigma; 1:5000 dilution) or rabbit anti-FLAG (PA1-984B from Invitrogen; 1:5000 dilution), or antibodies against myc (Santa Cruz 789; 1:5000), USP7 (Bethyl laboratories A300-033A; 1:10000), USP11 (Bethyl laboratories A301-613A; 1:5000), TRIP12 (Bethyl A301-814A; 1:2000), DDX24 (Bethyl A300-698A; 1:5000), DHX40 (ThermoFisher PA5-60685; 1:1000), PPM1G (Bethyl A300-881A; 1:5000), p53 (Santa Cruz sc-126; 1:1000) or actin (Santa Cruz 1615; 1:1000 dilution). Membranes were then washed three times in TBS-T, followed by incubation with goat anti-mouse HRP (Santa Cruz 2005) or goat anti-rabbit HRP (Sigma SAB3700878) at 1:5000 dilution. Membranes were developed using chemiluminescence reagents (ECL, Clarity ECL or ECL-prime; Santa Cruz, Bio-Rad or Amersham). Bands in Western blots were quantified using ImageQuantTL (GE healthcare Sciences),and normalized to actin bands.

### Immunoprecipitation

Cells transfected with the indicated mammalian expression plasmids were lysed on ice for 30 min in 4X volume of radioimmunoprecipitation assay (RIPA) buffer (50 mM Tris-HCl pH 8, 150 mM NaCl, 0.1% sodium deoxycholate, 0.5% NP-40) with complete protease inhibitors (Sigma P8340), followed by sonication and clarification by centrifugation. 4 mg of each clarified lysate was incubated with 20 μl of M2 anti-FLAG resin (Sigma) or anti-Myc resin (sc-40 AC Santa Cruz), overnight at 4 °C with end-over-end rotation. Resins were harvested by centrifugation, washed 4 times in lysis buffer, then boiled in 2x SDS loading buffer. Recovered proteins and 60 µg of the input were separated by SDS-PAGE and analyzed by Western blotting, as described above.

## Electronic supplementary material

Supplemental Figures

Supplementary Table S1

## Data Availability

All data generated or analysed during this study are included in this published article (and its Supplementary Information files).
