# Author Correction: Identification and Characterization of USP7 Targets in Cancer Cells

**DOI:** 10.1038/s41598-019-43448-4

**Published:** 2019-10-25

**Authors:** Anna Georges, Edyta Marcon, Jack Greenblatt, Lori Frappier

**Affiliations:** 1 Department of Molecular Genetics, University of Toronto, Toronto, Ontario Canada; 2 Donnelly Centre, University of Toronto, Toronto, Canada

Correction to: *Scientific Reports* 10.1038/s41598-018-34197-x, published online 26 October 2018

A supplementary file containing Table S1 was omitted from the original version of this Article. This has been corrected in the HTML version of the Article; the PDF version was correct at time of publication.

