# Supplementary material for: Identification and Characterization of USP7 Targets in Cancer Cells
Source: Sci Rep. 2018 Oct 26;8:15833. doi: 10.1038/s41598-018-34197-x (PMC6203733; doi:10.1038/s41598-018-34197-x)
Supplement: Supplementary file 1 — Supplemental Figures [file 41598_2018_34197_MOESM1_ESM.pdf]

Identification and Characterization of USP7 Targets in Cancer Cells

Anna Georges, Edyta Marcon, Jack Greenblatt and Lori Frappier

Supplementary Figures

Supplementary Figure S1. Uncropped Western blots

Figure 1A

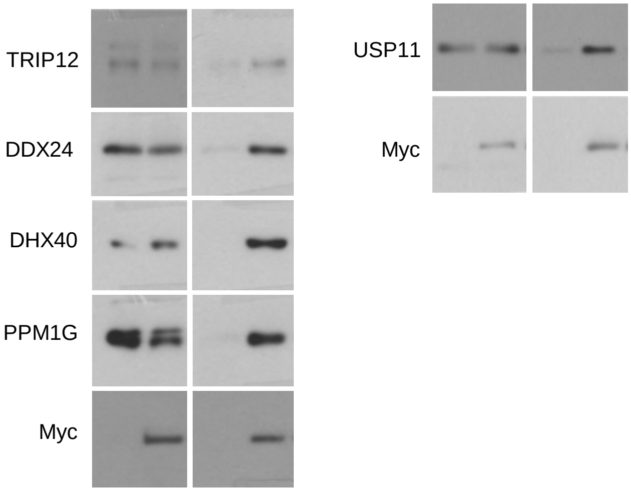

Figure 1B

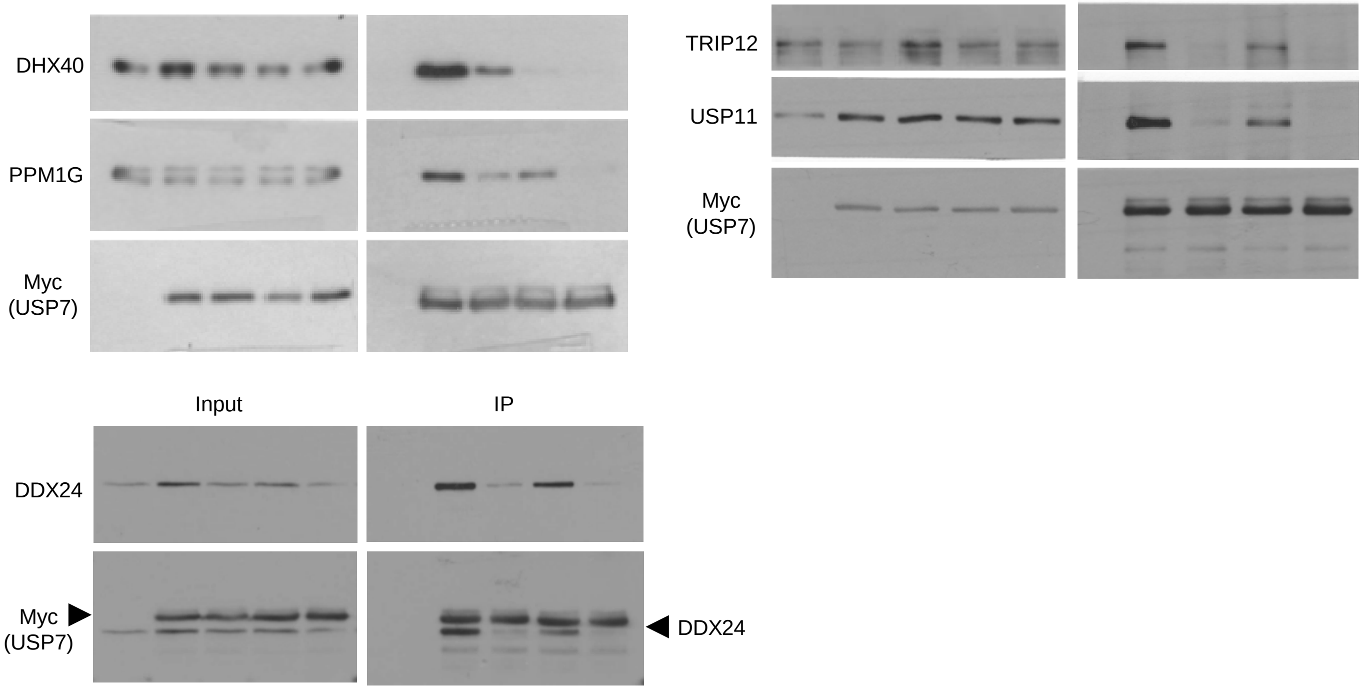

Figure 2B

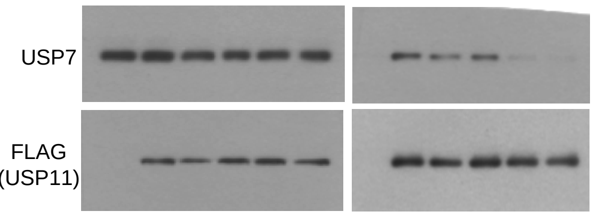

Figure 2C

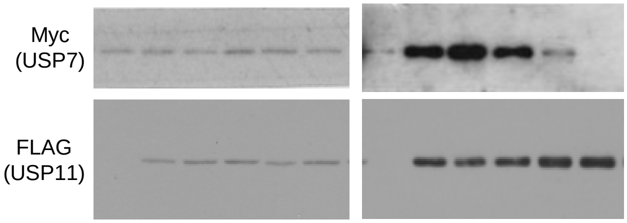

Figure 3B

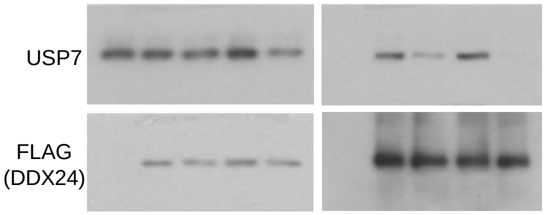

Figure 3C

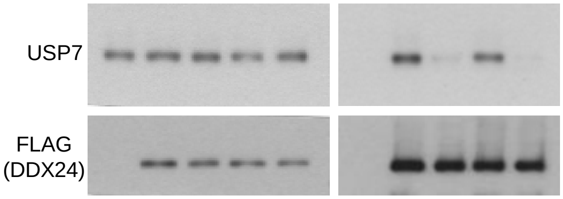

Figure 4A

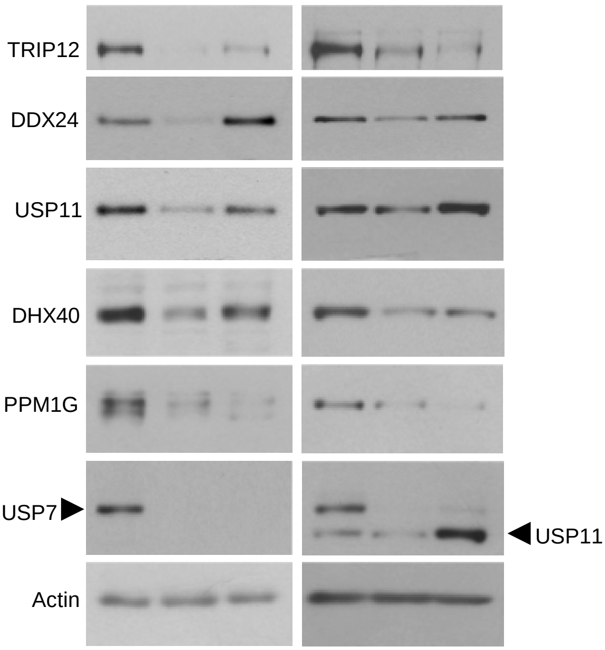

Figure 4B

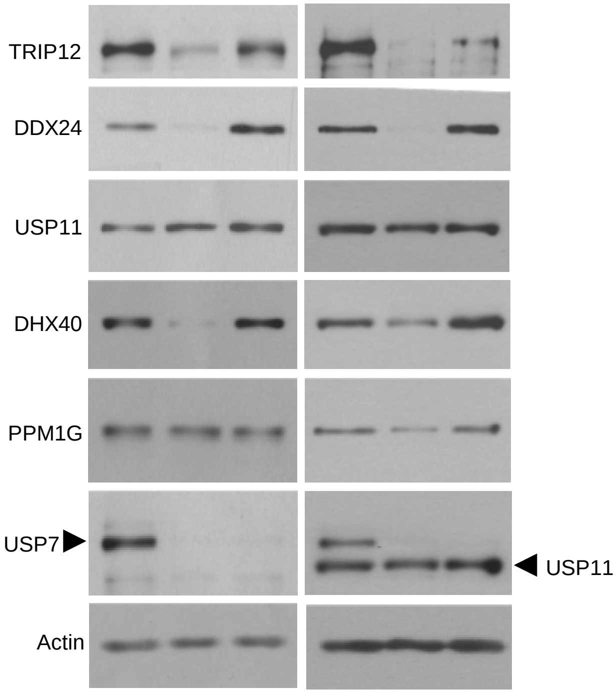

Figure 4C

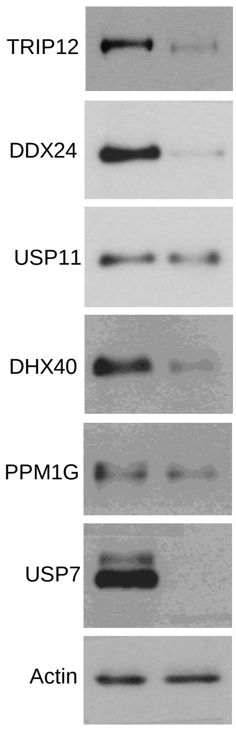

Figure 5A

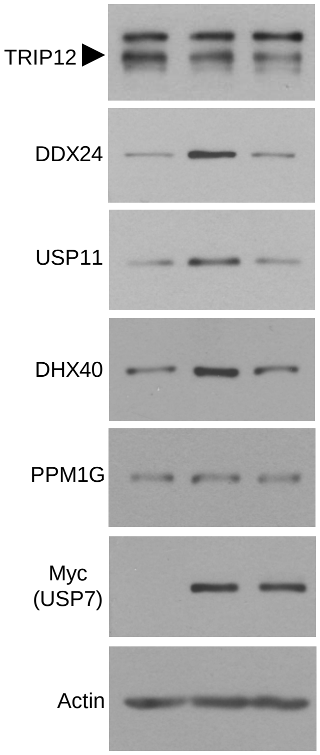

Figure 5B

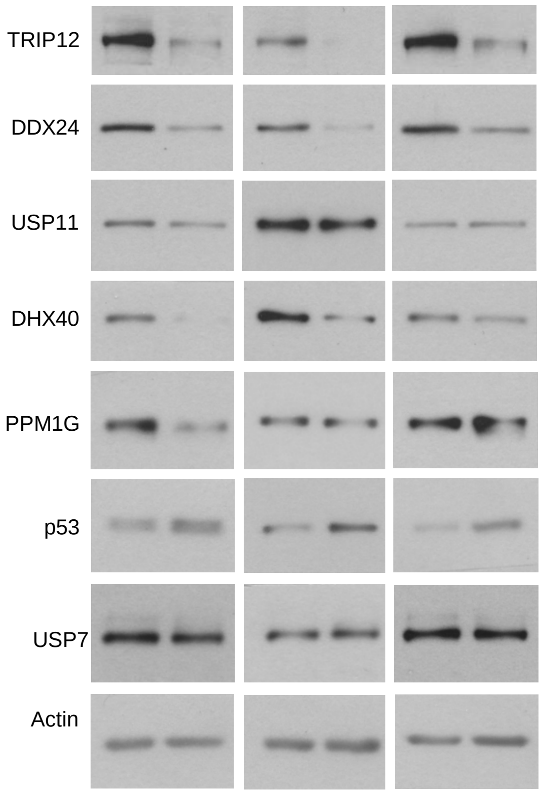

Supplementary Figure S1. Uncropped Western blots. Uncropped Western blots for each figure in the manuscript are shown as indicated. Note that the membranes themselves were cut into sections to enable probing of the same blots with multiple antibodies. Therefore the above membrane sections are the full blots for each antibody.

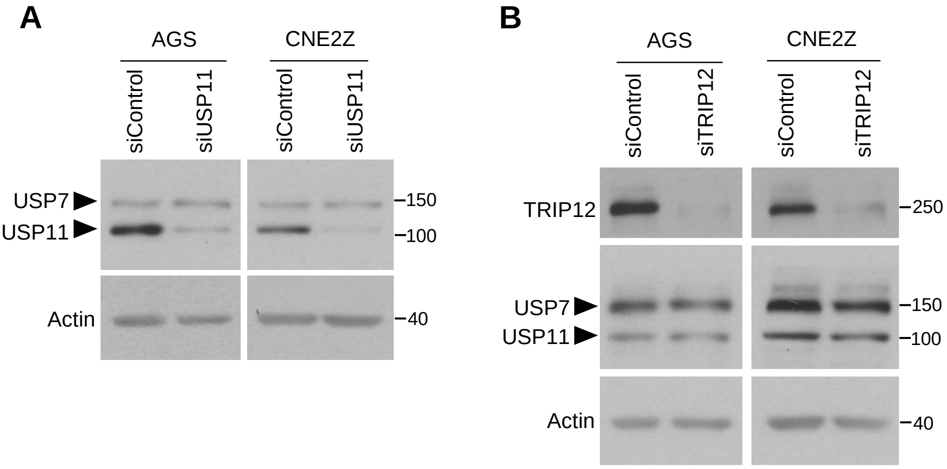

Supplementary Figure S2. **USP7 levels are not affected by silencing USP11 or TRIP12.** AGS or CNE2Z cells were transfected with siRNA targeting USP11 (A) or TRIP12 (B) or negative control siRNA. 48 hrs later cell lysates were analysed be Western blotting with antibodies against USP7, USP11 or TRIP12.
